# Comparative cytotoxicity and genotoxicity of commercial glyphosate-based herbicide formulations and co-formulants in human leukocyte and hepatocyte cell lines

**DOI:** 10.3389/ftox.2026.1770738

**Published:** 2026-07-03

**Authors:** Moustafa Sherif, Abderrahim Nemmar, Bassam R. Ali, Khadija Makame, Károly Nagy, Balázs Ádám

**Affiliations:** 1 Institute of Public Health, College of Medicine and Health Sciences, United Arab Emirates University, AI Ain, United Arab Emirates; 2 Department of Anatomy and Embryology, Benha University, Al-Qlayobia, Egypt; 3 Department of Physiology, College of Medicine and Health Sciences, United Arab Emirates University, AI Ain, United Arab Emirates; 4 Department of Genetics and Genomics, College of Medicine and Health Sciences, United Arab Emirates University, AI Ain, United Arab Emirates; 5 Department of Public Health and Epidemiology, Faculty of Medicine, University of Debrecen, Debrecen, Hungary

**Keywords:** comet assay, cytotoxicity, formulations, genotoxicity, glyphosate-based herbicides

## Abstract

**Background:**

Risk assessments usually test active ingredients but not full commercial formulations. We compared cytotoxic and genotoxic effects of three glyphosate-based herbicides (Roundup Mega, Glyfos, Fozat-480) and two co-formulants (ROKAmin SR22, EMPIGEN BB) in human HL60 (leukocyte) and HepG2 (hepatocyte) cells.

**Methods:**

Cells were exposed for 1 h to increasing concentrations (0.1–10,000 μM depending on formulation). Cytotoxicity was measured by propidium iodide staining; genotoxicity was assessed by the alkaline comet assay (tail DNA %, tail length, tail moment, Olive tail moment). Positive (100 μM H_2_O_2_) and negative controls were included. Data are means of three independent experiments.

**Results:**

Cytotoxicity occurred at lower concentrations in HL60 than HepG2. Roundup Mega and Glyfos produced the strongest genotoxic responses; Roundup Mega increased tail length in HL60 from 0.1 μM, while Glyfos produced consistent genotoxicity in HepG2 from 100 μM. Co-formulants alone showed limited genotoxicity, though ROKAmin SR22 induced DNA% in tail at higher concentrations. Genotoxic effects often occurred at sub-cytotoxic concentrations.

**Conclusion:**

Commercial GBH formulations can be more genotoxic than the active ingredient alone; formulation composition influences potency and target-cell sensitivity. These results support formulation-specific testing to improve human health risk assessment.

## Introduction

Pesticides represent one of the most extensively utilized chemical classes globally, with widespread applications in agriculture and non-agricultural settings despite mounting evidence linking long-term exposure to various adverse health effects, including chronic diseases in non-targeted organisms and humans (Darwish et al., 2026; Sherif et al., 2025; Sherif et al., 2026). The extensive use of these compounds has resulted in their ubiquitous presence in environmental matrices, with monitoring programs in the United States detecting pesticides in all sampled streams, more than 70% of common foods, and over half of adults and children (US Federal Insecticide, Fungicide, and Rodenticide Act FIFRA 2002). This pervasive exposure pattern underscores the critical need for comprehensive toxicological evaluation of pesticide products, particularly regarding their genotoxic and carcinogenic properties.

Glyphosate, an organophosphonate herbicide first registered in the United States in 1974, exemplifies the complexity surrounding pesticide safety assessment (USEPA, 2022). As one of the most widely used herbicides globally, glyphosate inhibits the enzyme 5-enolpyruvylshikimate-3-phosphate synthase (EPSPS), disrupting the shikimate pathway essential for aromatic amino acid biosynthesis in plants (PubChem, 2004). While glyphosate’s non-selective action and low soil mobility have contributed to its widespread adoption in agricultural settings, forestry, and urban landscaping, its safety profile remains subject to considerable scientific and regulatory debate.

The regulatory landscape surrounding glyphosate reflects this ongoing controversy. The World Health Organization’s International Agency for Research on Cancer (IARC) has classified glyphosate as “probably carcinogenic to humans,” while other regulatory bodies, including the U.S. Environmental Protection Agency (EPA), maintain that it is unlikely to pose carcinogenic risks when used according to guidelines. Similarly, glyphosate is currently approved as an active substance in the European Union until 15 December 2033, following assessments that concluded there was no scientific or legal basis for a ban under stipulated conditions and good agricultural practices (EC-Europa, 2017; EPA, 2025).

A fundamental limitation in current pesticide risk assessment lies in the predominant focus on active ingredients rather than complete commercial formulations (Mahmoud et al., 2026). In the United States, nearly 3,000 substances with varying toxicity levels are used as inert ingredients in pesticide products, with approximately 50% considered moderately risky. However, only a small proportion of toxicological tests required for pesticide registration include the complete pesticide formulation, while the majority examine only the active ingredient (Cox and Surgan, 2006). This approach may inadequately assess potential long-term effects, including significant concerns such as genetic damage, reproductive problems, and cancer (Ramadhan Makame et al., 2023).

Glyphosate-based herbicides (GBHs) exemplify this concern, as they contain proprietary “inert” ingredients whose identities and concentrations are often not fully disclosed that may be more toxic than glyphosate alone (Nagy et al., 2019). Commercial formulations such as Glyfos, Roundup Mega, and Fozat 480 commonly incorporate co-formulants, including surfactants like ROKAmin SR22 and EMPIGEN BB detergent, designed to enhance herbicidal activity through improved penetration and stability (Makame et al., 2025). However, these additives are suspected to exacerbate the herbicide’s toxic effects, raising concerns about the combined impact of glyphosate and its co-formulants on human health and the environment.

Genotoxicity assessment has emerged as a critical component in evaluating the potential human carcinogenicity of chemicals and understanding their mechanisms of action. Genotoxicity refers to the ability of chemicals, biological agents, and physical hazards to induce genetic damage in cells, either directly through binding to genetic material or indirectly through the generation of reactive oxygen species (ROS) that cause DNA strand breaks, abasic sites, and smaller molecular modifications. The human body possesses multiple protective mechanisms against genetic damage, including detoxifying enzymes such as paraoxonase 1 (PON-1), which play crucial roles in biotransforming toxic intermediates; and DNA repair enzymes that can recognize and correct various forms of DNA damage (Chatterjee and Walker, 2017); however, these protective mechanisms are not always sufficient, and metabolic processes can even increase toxicity in certain cases (Fang et al., 2012).

Recent studies investigating glyphosate-based herbicides using these genetic toxicology methods have revealed concerning patterns. Previous studies have already demonstrated that while glyphosate alone did not cause significant DNA damage or cell death, glyphosate-based herbicides induced both effects at concentrations of 250 μM and higher, with metabolic activation further increasing DNA damage induced by some GBHs (Nagy et al., 2021; Nagy et al., 2019). These findings suggest that the genotoxic potential of glyphosate-based complete commercial formulations may significantly exceed that of the active ingredient alone, necessitating comprehensive evaluation of both formulations and individual components to ensure accurate risk assessment and protect human health. Consistent with this view, a recent study evaluated genotoxicity of pure glyphosate and three commercial formulations (FAENA, TACKLE, CENTELLA) in human blood lymphocytes using the alkaline comet assay, demonstrating that all formulations induced genotoxic damage at all tested concentrations, and that FAENA and TACKLE produced significantly greater DNA migration than pure glyphosate alone—with each formulation displaying a characteristic damage pattern attributable to its distinct adjuvant composition (Alvarez-Moya and Reynoso-Silva, 2023). The present investigation extends this approach by employing two distinct human cell lines (HL60 and HepG2) representing separate target tissues, and by including isolated co-formulants, as well as full commercial formulations across a much wider, environmentally relevant concentration range (0.1–1000 μM), thereby enabling cell-type-specific and formulation-component-specific comparisons not possible in the earlier work.

## Methods

### Chemicals and formulations

Three commercially available glyphosate-based herbicide formulations were selected for this comparative investigation based on three criteria: (i) documented widespread use in agriculture across the UAE and the broader Middle East/European region; (ii) different co-formulant compositions—specifically the type and concentration of surfactant used —, which allowed systematic comparison of formulation effects independent of the shared active ingredient; and (iii) prior inclusion in genotoxicity studies by our group using a different endpoint (micronucleus assay), enabling cross-assay validation (Nagy et al., 2021; Nagy et al., 2019). The two co-formulants (ROKAmin SR22 and EMPIGEN BB) were selected because they are constituent adjuvants of the tested formulations and represent structurally distinct surfactant classes (ethoxylated amine versus betaine), permitting assessment of class-specific effects.

Roundup Mega, manufactured by Bayer Agriculture BV, contains 450 g/L glyphosate (CAS No: 70,901–12-1) as the potassium salt at 551 g/L (42% w/w), along with 7% (w/w) ethoxylated etheralkylamine (CAS No: 68,478–96-6). Glyfos, produced by Headland Agrochemicals Ltd., is formulated with 360 g/L (41% w/w) glyphosate as its isopropylamine salt and 9% w/w tallow alkyl amine ethoxylate. Fozat-480, manufactured by AGRO-CHEMIE Kft., contains 480 g/L glyphosate formulated as 41% N-(phosphonomethyl)glycine compound (CAS No: 38,641–94-0) with 2-propylamine (1:1) and less than 4% wetting agents.

Two co-formulants commonly used in glyphosate-based formulations were also investigated. ROKAmin SR22, a solid compound from PCC Exol SA, consists of C16-18 alkyl ethoxylated amines (CAS No. 61791–26-2) with a density of 1024 g/cm^3^ and an approximate molecular weight of 1250 g/mol. The compound exhibits good water solubility and a solidification point below 30 °C. EMPIGEN BB detergent (Product Number: 30,326, Sigma, Merck Life Science Kft.) is a 30% aqueous solution containing C12-14-alkyldimethyl-betaines (CAS No.: 66,455–29-6) as active substances, with a molecular weight of 271.44 g/mol.

### Cell culture

Two human cell lines were employed as *in vitro* models representing the primary target tissues of pesticide genotoxicity. The human Caucasian promyelocytic leukemia cell line (HL-60) was obtained through collaboration with Dr. Mahmood Al Mashhadani at the College of Medicine, Mohammed Bin Rashid University of Medicine and Health Sciences, Dubai, UAE. The original source of this cell line is AddexBio (San Diego, CA, USA; Catalogue #C0003023). The human hepatocellular carcinoma cell line (HepG2) was obtained through collaboration with Professor Bassam R. Ali (a co-author) at the College of Medicine and Health Sciences, United Arab Emirates University, whose laboratory originally purchased the cells from the American Type Culture Collection (ATCC, Manassas, VA, USA; Catalogue # HB-8065). Both collaborating laboratories acquired the cells directly from the respective repositories and shared them with our research team under active intra-institutional collaborations, consistent with the original providers’ Material Transfer Agreement terms. No primary human samples or human participants were used in this study. Therefore, this research did not require institutional human subjects ethical approval.

HL60 and HepG2 cell lines were obtained at low passage numbers. No primary human samples or human participants were used in this study. Therefore, this research did not require institutional human subjects ethical approval. Cell lines were tested for *mycoplasma* contamination (MycoAlert or PCR upon arrival to our lab) and were negative. Short tandem repeat (STR) profiling was not performed, as both cell lines were obtained directly from authenticated institutional repositories at low passage numbers and used within a limited passage range throughout the study.

Both cell lines were maintained in RPMI 1640 medium (Cat#: 11875093, Gibco) supplemented with 10% fetal bovine serum (Cat#: A5256701, Gibco) and 1% Penicillin-Streptomycin (10,000 U/mL) (Cat#: 15140122, Gibco). The HL60 cells were cultured as floating cells in suspension, while HepG2 cells were grown as an adherent monolayer. All cultures received fresh medium 24 h before experimentation to maintain optimal cell conditions.

### Cell treatment protocol

Stock solutions were prepared in RPMI medium for all test substances. For Roundup Mega, 37.9 μL was diluted in 10 mL of RPMI medium to achieve a 10,000 μM stock solution based on glyphosate’s molecular weight of 207.16 g/mol. Glyfos and Fozat-480 stock solutions were prepared by diluting 47 μL and 47.5 μL, respectively, in final volume of 10 mL of RPMI medium, considering the molecular weights of their respective glyphosate salts (228.19 g/mol and 228.18 g/mol). For ROKAmin SR22, a primary stock solution (9% v/v) was prepared by dissolving 0.9 mL in 9.1 mL of RPMI, from which 47 μL was further diluted in RPMI to obtain the working concentration. EMPIGEN BB detergent stock solution was prepared by dissolving 1.6 mL in 9.3 mL of RPMI, with subsequent dilution of 47.5 μL in RPMI for the final working solution. Serial dilutions were performed to obtain final test concentrations ranging from 0.1 to 1,500 μM for the GBHS’ formulations and co-formulants, with EMPIGEN BB detergent tested up to 10,000 μM. Detailed composition data for each formulation were obtained from their respective material safety data sheets.

Cells were exposed to increasing concentrations of test chemicals in cell culture medium for a duration of 1 hour at 37 °C. The 1-h exposure duration was selected to (i) maintain consistency with our prior publications using the same experimental system (Makame et al., 2025; Nagy et al., 2021; Nagy et al., 2019) enabling direct cross-study comparison; (ii) minimize confounding by cell proliferation and DNA repair, which can mask primary genotoxic events at longer time points; and (iii) reflect the acute exposure scenario relevant to occupational dermal or inhalation contact during pesticide application. This duration is established in the genotoxicity literature that detected significant GBH-induced DNA damage using comparable 1–4 h exposure windows in human cell lines (Gasnier et al., 2009; Koller et al., 2012).

The selection of test concentrations followed a two-step approach. First, a broad preliminary cytotoxicity screen was conducted across 0.1–10,000 μM in both cell lines to identify the concentration range causing ≥20% reduction in viability, consistent with OECD Test Guideline 487. Second, concentrations for genotoxicity testing were restricted to those maintaining ≥80% cell viability to ensure that observed DNA damage reflected genotoxic action rather than secondary effects of cytotoxicity. The lower end of the concentration range (0.1–10 μM) was selected to cover low-to-moderate exposure scenarios. In Acquavella et al. (2004), urinary glyphosate in farmers was reported at 3 ppb on average and up to 233 ppb at the maximum, which corresponds to approximately 0.02–1.38 μM. Based on cytotoxicity results, test concentrations for genotoxicity were cell line-specific as detailed in Table 1.

**TABLE 1:** Stock solution preparation and treatment concentrations.

Test substance	Stock concentration	Stock volume	Final volume	HL60 treatment concentrations (μM) for genotoxicity	HepG2 treatment concentrations (μM) for genotoxicity
Glyphosate-based herbicides					
Roundup mega	10,000 μM	37.9 μL	10 mL	0, 0.1, 1, 10, 50, 100, 150, 200	0, 0.1, 1, 10, 50, 100, 200, 500, 1000
Glyfos	10,000 μM	47.0 μL	10 mL	0, 0.1, 1, 10, 50, 100	0, 0.1, 1, 10, 50, 100, 200, 500, 1000
Fozat-480	10,000 μM	47.5 μL	10 mL	0, 0.1, 1, 10, 50, 100, 150, 200	0, 0.1, 1, 10, 50, 100, 200, 500, 1000
Co-formulants					
ROKAmin SR22	10,000 μM*	47.0 μL***	10 mL	0, 0.1, 1, 10, 50, 100, 150, 200	0, 0.1, 1, 10, 50, 100, 200, 500, 1000
EMPIGEN BB	10,000 μM**	47.5 μL***	10 mL	0, 0.1, 1, 10, 50, 100, 150, 200	0, 0.1, 1, 10, 50, 100, 200, 500, 1000

* Primary stock solution (9% v/v) prepared by dissolving 0.9 mL ROKAmin SR22 in 9.1 mL RPMI medium.

** Primary stock solution prepared by dissolving 1.6 mL EMPIGEN BB in 9.3 mL of RPMI RPMI medium

*** Volume taken from primary stock solution for further dilution.

### Cytotoxicity assessment

Cell viability was determined using propidium iodide (PI) fluorescent labeling immediately following treatment. PI exclusion test was selected over metabolic assays (e.g., MTT, MTS) because HL60 cells grow in suspension, making the washing and formazan solubilization steps of tetrazolium-based assays technically unreliable and prone to artifactual variation. PI-based flow cytometric or microscopic enumeration provides a direct, membrane-integrity-based measure of cell death that is equally valid for both suspension and adherent cells and is specifically recommended for suspension cell lines in genotoxicity testing protocols (Tice et al., 2000). PI, a DNA intercalating dye, selectively penetrates the membranes of deceased and dying cells while being excluded by the intact plasma membranes of viable cells. The PI fluorescent dye was dissolved in PBS (pH 7.2) to achieve a final concentration of 2 μM. Subsequently, 200 μL of this working solution was added to 1 × 10^5^ cells and incubated for 30 min at 4 °C in darkness. Following labeling, cells were washed and resuspended in ice-cold PBS buffer. A 40 μL aliquot of cell suspension was placed on a microscope slide for immediate examination under a Zeiss epifluorescent microscope at ×20 magnification. Total cell counts were determined using transmitted light microscopy on two non-overlapping fields per slide (minimum 200 cells per concentration per experiment), while PI-labeled cells were visualized using the TRITC filter. Cell viability percentage was calculated by subtracting the count of PI fluorescent cells from the total cell count. The mean percentage of living cells from repeated experiments represented the final cell viability.

### Genotoxicity analysis

DNA damage was assessed using the alkaline version of the comet assay, which was selected as the primary genotoxicity endpoint because it detects a broad spectrum of DNA lesions—including single- and double-strand breaks and alkali-labile sites—at the single-cell level, with high sensitivity at sub-cytotoxic concentrations (Collins, 2004; Tice et al., 2000). This method is endorsed by the OECD (Test Guideline 489) and has been the most widely applied *in vitro* genotoxicity assay used to test pesticide formulations, enabling direct comparison with the existing literature (OECD, 2016). Following treatment, samples were centrifuged and cells were resuspended in PBS at a density of 1800 cells/μL. Frosted degreased slides were coated with a single layer of 1% normal melting point agarose overlaid with 0.75% low melting point agarose containing 2 × 10^5^ cells per slide. A 20 × 20 mm coverslip was applied to ensure proper spreading of the cell-agarose mixture across the slide surface.

After 10 min of solidification at 4 °C, coverslips were removed and embedded cells underwent prolonged lysis using CometAssay Lysis Solution (Cat#4250–050-01) overnight at 4 °C in darkness. Subsequently, DNA was allowed to unwind for 20 min in alkaline unwinding solution (300 mM NaOH, 1 mM EDTA) and then subjected to electrophoresis in the same solution for 20 min at 1 V/cm and 300 mA. Slides were gently rinsed twice with Milli-Q water to eliminate residual alkali and detergent, followed by two washes with 70% ethanol. After drying for 10 min in darkness, each slide was stained with PI (20 μg/mL), washed with water for 1 minute, covered with a coverslip, and stored in a humidified container until analysis.

Fluorescence signals were captured at ×40 magnification using the Metafer automated image analysis system from MetaSystems GmbH, Germany, equipped with ZEISS Axio Imager Z2 and cometscan module. The Neon Software (MetaSystems GmbH, Germany) performed the analysis of more than 100 randomly captured comet images from duplicate slides per concentration and the computation of DNA damage parameters. Four DNA damage parameters were quantified: tail DNA percentage, tail length (μm), tail moment, and Olive tail moment. Representative comet image is presented in Supplementary Figure S1 to illustrate the DNA migration patterns detected in the Metafer system. Tail DNA percentage measures the quantity of DNA in the tail, tail length indicates the migration distance of DNA from the nucleus, tail moment represents the product of tail length and the proportion of DNA in the tail, and Olive tail moment is the product of tail DNA percentage and the distance between the centers of mass of the head and the tail.

### Statistical analysis

All experiments were performed independently three times alongside 100 µM H_2_O_2_-treated positive and untreated negative controls, as displayed in Supplementary Figure S1. Cell viability was calculated as one minus cytotoxicity, represented by the mean proportion of living cells from three repetitions. For comet analysis, only slides containing more than 100 images per concentration for each experiment were considered, with slides containing fewer images discarded from the analysis.

The median values of comet assay metrics (percentage of DNA in the tail, tail length, tail moment, Olive tail moment) at various concentrations of test chemicals were calculated using Microsoft Excel 2021 (eliminating within-experiment positive skewness inherent to comet data distributions), and the arithmetic mean of the medians from the repeated independent experiments was considered in the statistical analysis for group comparison. The two-step approach that substantially normalises the distribution of values submitted to parametric testing is consistent with established comet assay statistical practice (OECD, 2016). The arithmetic mean of cell viability and comet assay metrics at various concentrations were compared to those of untreated cells using analysis of variance (ANOVA) followed by Dunnett’s post hoc test, which appropriately controls the family-wise error rate when multiple treatment groups are compared against a single control. The analyses were performed using RStudio (version 2025.09.2 + 418, Cucumberleaf Sunflower; Posit PBC, Boston, MA, USA) with the following packages: and readxl (v1.4.3) for data import, ggplot2 (v3.5.1) for figure generation, multcomp (v1.4–26) for Dunnett’s post hoc contrasts; all packages were sourced from CRAN (https://cran.r-project.org). Statistical significance was determined at 5% significance level. Results were presented as the mean of the median values of DNA damage parameters from three independent experiments. Error bars in figures represent ±1 standard error to illustrate the variability of data across different experiments. The overall experimental design and presentation approach aligns with established practices for *in vitro* cytotoxicity and genotoxicity assessment in human cell lines.

## Results

### Toxicological assessments in leukocytes

**FIGURE 1:**
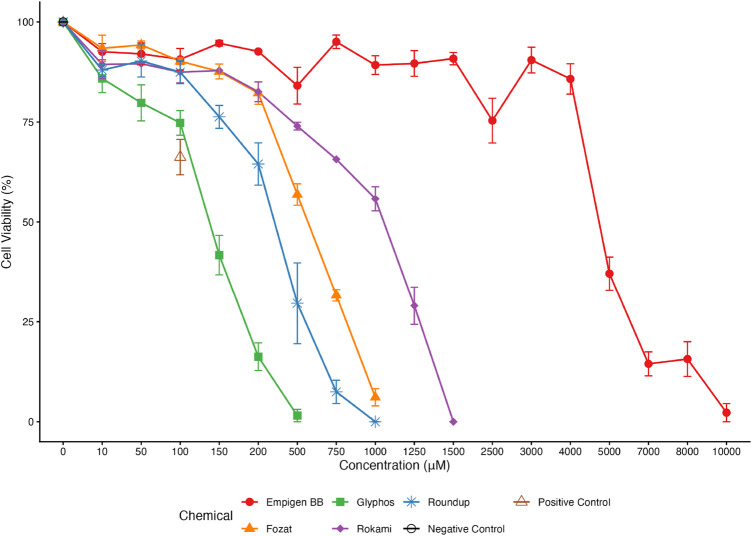
Cytotoxicity of glyphosate-based formulations and co-formulants in HL60 cells. Cell viability (%) after 1-hour exposure to different concentrations of glyphosate-based formulations (Roundup Mega, Glyfos, Fozat 480) and co-formulants (EMPIGEN BB detergent, ROKAmin SR22) in the 0-10000 μM range. Data points represent means ± standard error from at least three independent experiments. Asterisks indicating statistically significant differences compared to untreated control (*p < 0.05, **p < 0.01, ***p < 0.001) are presented in Supplemental Table S1.

Cytotoxicity was evaluated in a concentration range of 0–10,000 µM. The cytotoxicity assessment revealed concentration-dependent decreases in cell viability for all tested formulations (Figure 1). Glyfos and ROKAmin SR22 demonstrated significant cytotoxicity at the lowest concentration tested (10 μM), with mean differences (MDs) of −14.112 ± 4.814 (p = 0.024) and −10.621 ± 3.676 (p = 0.029), respectively. Fozat 480 showed significant cytotoxicity at 100 μM (MD = −9.814 ± 2.898, p = 0.010), while Roundup Mega at 150 μM (MD = −23.714 ± 6.219, p = 0.004). Embigen BB required the highest concentration to induce significant cytotoxicity at 500 μM (MD = −15.916 ± 4.317, p = 0.005).

The glyphosate-based formulations produced the steepest decline in cell viability, with complete cell death occurring at 500 μM and 1000 μM. The co-formulant ROKAmin SR22 demonstrated a more gradual decline, with complete cell death at approximately 1500 μM, while Embigen BB exhibited the weakest cytotoxic profile, with viability over 80% maintained up to 5000 μM, followed by a steep decline at higher concentrations.

**FIGURE 2:**
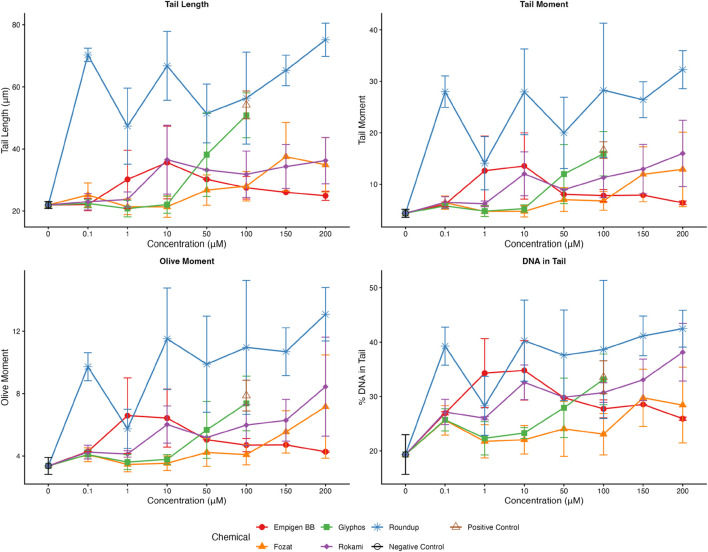
Genotoxic effects of glyphosate-based formulations and co-formulants in HL60 cells. The graphs show four comet assay parameters (DNA% in tail, tail length, tail moment, and Olive tail moment) measured after 1-h exposure to different concentrations of glyphosate-based formulations (Roundup Mega, Glyphos, Fozat 480) and co-formulants (Embigen BB, ROKAmin SR22) in the 0–200 μM range based on their cytotoxicity profile. Error bars represent standard error of the mean of three repeated experiments. Asterisks indicating statistically significant differences compared to untreated control (*p < 0.05, **p < 0.01, ***p < 0.001) are presented in Supplementary Table S1.

The genotoxicity dose-response curves demonstrated unique patterns for each chemical across the four DNA damage parameters in leukocytes as displayed in Figure 2.

For percentage of DNA in tail, significant increases were observed for Glyfos and ROKAmin SR22, while Roundup Mega, Fozat 480, and EMPIGEN BB detergent did not demonstrate statistically significant genotoxicity in this parameter. Glyfos induced significant DNA damage at 100 μM (MD = 13.817 ± 5.470, p = 0.038), representing the lowest concentration among the chemicals, while ROKAmin SR22 showed significant effect at 200 μM (MD = 18.799 ± 5.790, p = 0.015).

Tail length measurements revealed significant increases for Roundup Mega and Glyfos. The lowest effective concentration was observed with Roundup Mega at 0.1 μM (MD = 48.343 ± 14.904, p = 0.000), demonstrating its high potency in inducing DNA damage. Glyfos showed significant effect at 100 μM (MD = 28.868 ± 10.165, p = 0.021), while Fozat 480, ROKAmin SR22, and EMPIGEN BB detergent did not show statistically significant effects at the tested concentrations.

Tail moment also showed significant increases for Roundup Mega and Glyfos only. Glyfos induced significant effect at 100 μM (MD = 11.566 ± 4.697, p = 0.043), while Roundup Mega showed significance at 200 μM (MD = 27.883 ± 10.233, p = 0.033). Fozat 480, ROKAmin SR22, and EMPIGEN BB detergent did not demonstrate statistically significant effects at the tested concentrations.

The Olive tail moment parameter revealed significant genotoxicity only for Roundup Mega among the tested chemicals. It showed significant effect at 200 μM (MD = 9.724 ± 3.741, p = 0.042). Neither Glyfos, Fozat 480, ROKAmin SR22, or EMPIGEN BB detergent demonstrated statistically significant effects with Olive tail moment at the tested concentrations.

In summary, the results indicate that Roundup Mega is the most genotoxic among the tested glyphosate-based formulations in terms of general effect size, tail length and Olive tail moment, followed by Glyfos, which showed significant effects in both DNA% in tail and tail moment. Fozat did not demonstrate statistically significant genotoxic effects in any parameter. Regarding the co-formulants, only ROKAmin SR22 showed genotoxic effects and only with DNA% in tail, while both co-formulants showed cytotoxic potential, especially ROKAmin SR22.

### Toxicological assessments in hepatocytes

**FIGURE 3:**
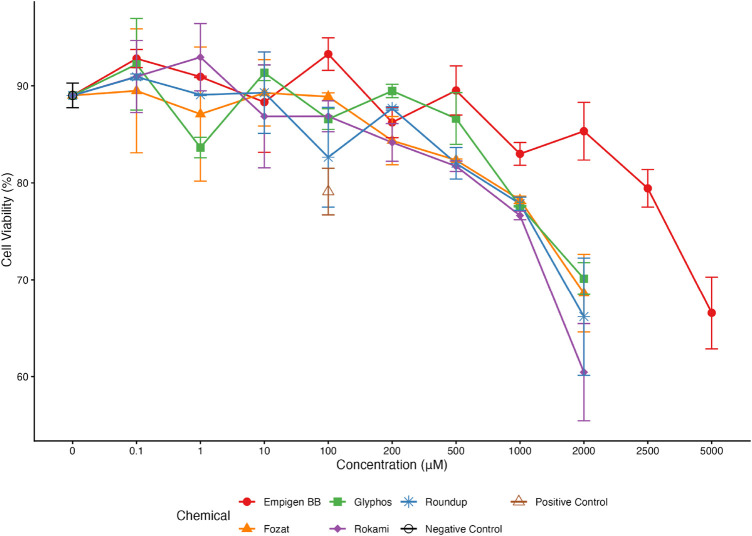
Cytotoxic effects of glyphosate-based formulations and co-formulants in HepG2 cells. The graph shows cell viability (%) of HepG2 cells following 1-hour exposure to various concentrations (0-5000 μM) of glyphosate-based formulations (Roundup Mega, Glyfos, Fozat 480) and co-formulants (EMPIGEN BB detergent, ROKAmin SR22). Error bars represent standard error of the mean of three repeated experiments are presented in Supplemental Table S3.

Cell viability assessed in HepG2 cells demonstrated a dose-dependent response of the tested chemicals (Figure 3). All formulations maintained viability above 80% at concentrations up to 500 μM, after which a gradual decline was observed. At higher concentrations, significant reductions in viability were observed for all chemicals. ROKAmin SR22 exhibited the strongest cytotoxic effect with viability decreasing significantly at 1000 μM (MD = −28.559 ± 4.368, p < 0.001). Glyfos demonstrated significant effects at 1000 μM (MD = −18.885 ± 2.807, p < 0.001), Roundup Mega at 2000 μM (MD = −22.825 ± 4.318, p = 0.001), while Fozat 480 at 2000 μM (MD = −20.397 ± 5.225, p = 0.010). EMPIGEN BB detergent showed significant cytotoxicity only at 5000 μM (MD = −22.439 ± 3.492, p < 0.001).

**FIGURE 4:**
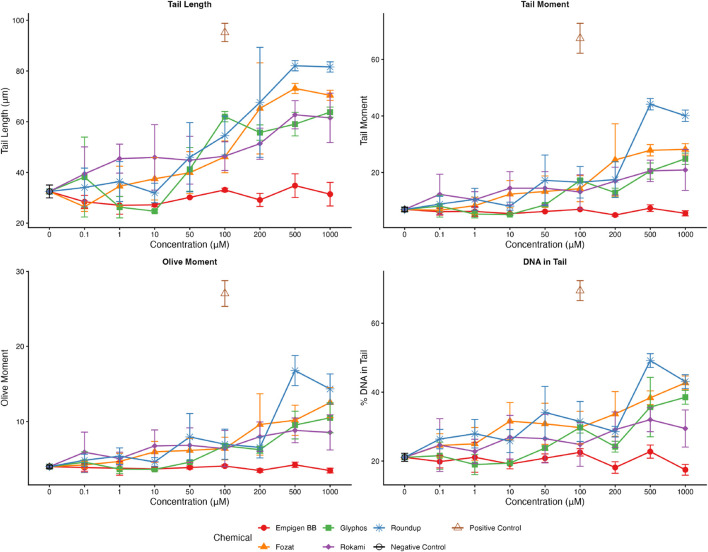
Genotoxic effects of glyphosate-based formulations and co-formulants in HepG2 cells. The graphs show four comet assay parameters (DNA% in tail, tail length, tail moment, and Olive tail moment) measured after 1-hour exposure to different concentrations of glyphosate-based formulations (Roundup Mega, Glyphos, Fozat 480) and co-formulants (ROKAmin SR22, EMPIGEN BB detergent) in the 0-1000 μM range based on their cytotoxicity profile. Error bars represent standard error of the mean of three repeated experiments. Asterisks indicating statistically significant differences compared to untreated control (*p < 0.05, **p < 0.01, ***p < 0.001) are presented in Supplemental Table S4.

The genotoxic potential of the tested chemicals in HepG2 cells demonstrated distinct dose-response as displayed in Figure 4.

Percentage of DNA in tail revealed significant increases for three of the five tested chemicals. Roundup Mega induced DNA damage from 500 μM (MD = 28.094 ± 8.113, p = 0.008), demonstrating the largest genotoxic effect among the tested formulations. Glyfos showed significant DNA damage also from 500 μM (MD = 14.589 ± 4.556, p = 0.022), while Fozat 480 at 1000 μM (MD = 21.621 ± 8.358, p = 0.045). Neither ROKAmin SR22 nor EMPIGEN BB detergent induced statistically significant increases in DNA% in tail at any tested concentration.

Glyfos induced significant increase of tail length from the lowest concentration of 100 μM (MD = 29.511 ± 7.804, p = 0.008). Both Roundup Mega and Fozat 480 showed significant increases from 500 μM (MD = 49.579 ± 15.613, p = 0.014 and MD = 40.652 ± 14.652, p = 0.032, respectively), Roundup Mega having the largest effect size among all test chemicals. Both ROKAmin SR22 and EMPIGEN BB detergent failed to induce statistically significant increases in tail length at any of the tested concentrations, although ROKAmin SR22 was near significance at 500 μM (MD = 30.248 ± 12.482, p = 0.056).

Glyfos induced significant tail moment increase from the lowest concentration of 100 μM (MD = 10.248 ± 2.260, p = 0.002), consistent with its effects on tail length. Roundup Mega demonstrated significant tail moment increase from 500 μM (MD = 37.232 ± 8.287, p < 0.001) with the largest effect size, while Fozat 480, ROKAmin SR22, and EMPIGEN BB detergent did not induce statistically significant changes in tail moment at any of the tested concentrations.

Glyfos produced significant Olive tail moment increase at the lowest concentration of 100 μM (MD = 2.769 ± 1.003, p = 0.046) and from 500 μM (MD = 5.538 ± 1.099, p = 0.001), again demonstrating early-onset DNA damage. Roundup Mega showed significant increases from 500 μM (MD = 12.77 ± 2.749, p < 0.001) with the largest effect, while Fozat 480 demonstrated significant effect at 1000 μM (MD = 8.525 ± 3.106, p = 0.034). Similar to other parameters, neither ROKAmin SR22 nor EMPIGEN BB detergent induced statistically significant increases in Olive tail moment across the concentration range tested.

Among all tested formulations, Glyfos consistently demonstrated genotoxic effects at the lowest concentration (100 μM) across three parameters, while Roundup Mega exhibited the largest genotoxic effects from 500 μM across all four parameters. Fozat 480 showed moderate genotoxicity at concentrations of 500–1000 μM. Despite their cytotoxic potential, ROKAmin SR22 and EMPIGEN BB detergent did not induce statistically significant genotoxic effects in any of the measured parameters in the tested concentration range.

**FIGURE 5:**
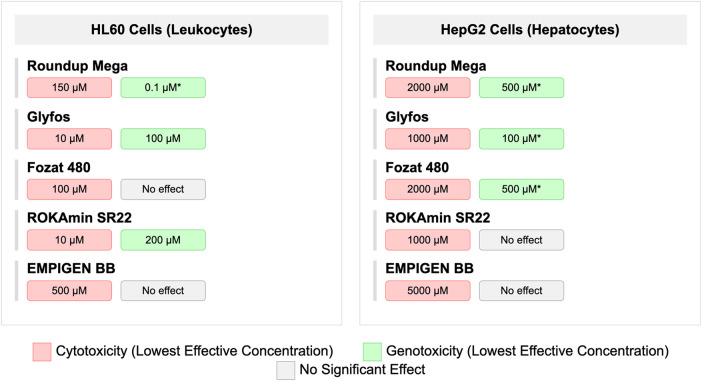
Comparative summary of the lowest effective concentrations (μM) for cytotoxicity and genotoxicity of glyphosate-based herbicide formulations and co-formulants in HL60 and HepG2 cell lines. Values represent the lowest concentration showing statistically significant effects (p < 0.05) compared to untreated controls after 1-hour exposure. *Indicates genotoxic effects occurring at concentrations below cytotoxic thresholds.

To provide a comprehensive overview of the differential toxicity profiles, Figure 5 summarizes the lowest effective concentrations for both cytotoxic and genotoxic effects across all tested substances and both cell lines, clearly demonstrating the occurrence of DNA damage at sub-cytotoxic concentrations for several formulations.

## Discussion

This comprehensive assessment of cytotoxicity and genotoxicity in HL60 and HepG2 cells contributes to the ongoing scientific and regulatory discourse surrounding glyphosate safety (EFSA, 2017; IARC, 2015). The divergence between regulatory agencies—with IARC classifying glyphosate as “probably carcinogenic” while EFSA and EPA did not find sufficient evidence—underscores the importance of investigating not only the active ingredient but also complete commercial formulations and their co-formulants to comprehensively assess health risks.

Cytotoxicity profiles differed markedly between cell lines and across compounds. In HL60 cells, thresholds ranged from 10 μM (Glyfos, ROKAmin SR22) to 500 μM (EMPIGEN BB), reflecting variability in membrane disruption capacity attributable to the differing surfactant chemistry of each formulation rather than their shared N-(phosphonomethyl)glycine active ingredient. In HepG2 cells, all substances maintained viability above 80% up to 500 μM, with significant declines emerging at 1,000–2,000 μM—consistent with the greater metabolic detoxification capacity of hepatocytes (Furukawa et al., 2004). These cell-line differences align with Nagy et al.'s findings in human mononuclear white blood cells, where pure glyphosate showed limited cytotoxicity even at 10,000 μM, while commercial GBH formulations induced significant cell death from 1,000 μM regardless of metabolic activation, implicating non-active ingredients as primary drivers of cytotoxic potential (Nagy et al., 2021). This is further supported by Mesnage et al.'s systematic review demonstrating that commercial GBH formulations are consistently more toxic than glyphosate alone across mammalian species, with enhanced toxicity attributed to adjuvants that act independently and, in some cases, facilitate the uptake of environmental contaminants present in or alongside commercial formulations (Mesnage et al., 2015).

The genotoxicity findings revealed patterns that varied by cell type and chemical formulation. In HL60 cells, Roundup Mega demonstrated remarkable genotoxic potential across all parameters, especially in tail length, inducing statistically significant DNA damage at concentrations as low as 0.1 μM—well below cytotoxic thresholds —, suggesting direct DNA-damaging mechanisms independent of cell death. Glyfos showed significant genotoxicity at 100 μM across DNA% in tail and tail length, while Fozat 480 demonstrated no significant genotoxic effects in HL60 cells despite its cytotoxic activity, indicating a dissociation between cytotoxic and genotoxic mechanisms. These formulation-specific differences align with Nagy et al.'s findings that commercial GBH formulations—including the same products tested here—exhibited significantly enhanced genotoxicity relative to pure glyphosate alone, which showed minimal DNA damage across tested concentrations. The sensitivity differential between endpoints is notable: Nagy et al. detected Glyfos- and Fozat 480-induced micronucleus formation at 10 μM in peripheral blood mononuclear cells, whereas our comet assay identified Glyfos genotoxicity only from 100 μM and found no significant effect for Fozat 480 in HL60 cells—illustrating that assay type and cell model jointly determine the detectable genotoxic threshold, and reinforcing the importance of employing multiple endpoints in comprehensive genotoxicity assessment (Nagy et al., 2021; Nagy et al., 2019). Importantly, the absence of significant S9-mediated enhancement of genotoxicity reported by Nagy et al. further supports a direct DNA-damaging mechanism for these formulations rather than metabolite-mediated toxicity. Alvarez-Moya and Reynoso-Silva (2023) found that two of three tested commercial GBH formulations (FAENA and TACKLE) produced greater DNA migration than pure glyphosate in human lymphocytes, while a third (CENTELLA) induced a qualitatively distinct damage pattern—collectively illustrating that adjuvant identity, rather than active ingredient concentration, is a primary determinant of genotoxic outcome (Alvarez-Moya and Reynoso-Silva, 2023). This principle is further supported by several independent lines of evidence: Roundup Mega induced DNA damage in liver and blood cells at concentrations where glyphosate alone had no effect (Mañas et al., 2009); Roundup formulations induced DNA migration in buccal epithelial cells at concentrations 50-fold lower than required for glyphosate alone (Koller et al., 2012); and even formulations lacking conventional surfactants caused significant DNA damage (Benachour and Seralini, 2009; Haefs et al., 2002). Bolognesi et al. (1997) similarly showed that Roundup Mega consistently demonstrated higher micronucleus-inducing potential than other formulations and pure glyphosate at equivalent concentrations (Bolognesi et al., 1997).

The capacity of GBH formulations to cause DNA damage at sub-cytotoxic concentrations—observed consistently across these studies and in our own data—emphasises the need for independent genotoxicity assessment decoupled from cytotoxicity, a principle endorsed by current regulatory frameworks (Basketter et al., 2012; OECD, 2016). A consistent methodological observation across both cell lines in our study was the high sensitivity of tail length as a genotoxic marker, despite DNA% in tail being the most commonly reported comet parameter (Collins, 2004; Tice et al., 2000), underscoring the value of reporting all four comet assay parameters as recommended by OECD guidelines. At the population level, the genotoxic effects observed in laboratory GBH studies are increasingly corroborated by meta-analytical evidence linking glyphosate exposure to non-Hodgkin lymphoma risk (Schinasi and Leon, 2014; Zhang et al., 2019), suggesting translational relevance to real-world health outcomes.

Regarding the co-formulants, ROKAmin SR22 showed significant genotoxicity only for the DNA% in tail parameter at 200 μM in HL60 cells, with no significant effects on tail length, tail moment, or Olive tail moment, while EMPIGEN BB detergent did not induce statistically significant genotoxic effects in any parameter across the tested concentration range. These findings suggest that co-formulants alone contribute limited direct genotoxic activity, though they differ in relative potency. This is consistent with variable genotoxicity profiles reported for structurally related surfactants—some of which show minimal DNA damage across multiple assays, while others, such as Roksol TL-7, produce positive results in *Salmonella* reversion and sister chromatid exchange induction tests (Janik-Spiechowicz et al., 1992). The relative potency order observed here—ROKAmin SR22 > EMPIGEN BB—is corroborated by Makame et al. (2025), who found that ROKAmin SR22 induced micronucleus formation at concentrations as low as 1 μM in human mononuclear white blood cells, whereas EMPIGEN BB required 100 μM under the same conditions (Makame et al., 2025). Notably, that study also found that the addition of the S9 metabolic fraction abolished genotoxicity for both co-formulants and the full GBH formulations, which aligns with our observation that HepG2 cells—with their greater phase I and II metabolic capacity—show reduced sensitivity to these genotoxic insults compared to HL60 cells, and points to biotransformation as a key modulator of co-formulant-mediated DNA damage.

The differential sensitivity between cell lines reflects fundamental differences in their biochemical makeup. HL60 is a p53-deficient promyelocytic leukemia line with impaired cell cycle checkpoint function, rendering it highly susceptible to genotoxic stress and producing elevated background DNA migration in the comet assay (Wolf and Rotter, 1985). Against this elevated baseline, significant increases in DNA% in tail were detected only for Glyfos and ROKAmin SR22 in HL60 cells. HepG2 cells, while retaining partial CYP expression and phase II conjugation capacity, have reduced xenobiotic-metabolising activity relative to primary hepatocytes (Arzumanian et al., 2021); in this line, oxidative stress has been identified as a key mechanistic driver of pesticide-induced DNA strand breaks, as demonstrated for cypermethrin (AlKahtane et al., 2018). These cell-type-specific characteristics set the mechanistic context for the genotoxicity patterns described in the following sections.

In HepG2 cells, the genotoxicity profiles demonstrated distinct patterns from those observed in HL60 cells. Glyfos consistently induced genotoxic effects at the lowest concentration (100 μM) across three parameters (tail length, tail moment, and Olive tail moment), with an earlier onset than observed in HL60 cells, while Roundup Mega exhibited the largest overall effect size from 500 μM across all four parameters. Fozat 480, which showed no significant genotoxicity in HL60 cells, demonstrated moderate genotoxic effects in HepG2 cells from 500 μM (tail length) and 1000 μM (DNA% in tail and Olive tail moment) — a cell-type-specific response that likely reflects hepatocyte-specific metabolic transformation generating reactive intermediates capable of causing DNA strand breaks, a pathway that would be absent in the metabolically limited HL60 cell line. Despite their cytotoxic potential at higher concentrations, neither ROKAmin SR22 nor EMPIGEN BB induced statistically significant genotoxic effects in any parameter in HepG2 cells across the tested range. These HepG2 findings are consistent with prior hepatocyte model data: Gasnier et al. (2009) detected comet assay DNA damage in HepG2 cells from approximately 30 μM glyphosate-equivalent of four Roundup formulations, while glyphosate alone produced no genotoxic effect (Gasnier et al., 2009); Kašuba et al. (2017) reported significant micronucleus formation at concentrations as low as 2.9 μM after 4-h exposure (Kašuba et al., 2017); and Chaufan et al. (2014) demonstrated that Roundup UltraMax caused cytotoxic and oxidative effects in HepG2 cells at concentrations where pure glyphosate and its metabolite AMPA were inactive (Chaufan et al., 2014). Collectively, these studies and our data converge on the conclusion that the genotoxic and cytotoxic activity of commercial GBH formulations in hepatocyte models is driven by formulation composition rather than glyphosate itself, with current regulatory thresholds—based predominantly on the active ingredient—potentially insufficient to protect against the full damage potential of commercial products (Cox and Surgan, 2006; Ramadhan Makame et al., 2023).

The mechanistic basis of the observed formulation toxicity is most plausibly linked to oxidative stress. Our companion publication (Makame et al., 2023) demonstrated that GBHs and their co-formulants significantly reduced superoxide dismutase (SOD) activity in human mononuclear white blood cells—an effect not seen with pure glyphosate—indicating that adjuvants drive cytotoxicity through oxidative stress induction. This is corroborated by Gasnier et al. (2009), who showed that Roundup formulations altered endocrine-related endpoints alongside DNA damage in HepG2 cells, consistent with ROS-mediated cellular disruption (Gasnier et al., 2009). Together, these findings suggest that oxidative damage is a primary pathway through which co-formulants amplify the toxicity of commercial GBH products, although direct ROS quantification and mechanism-specific follow-up in the current cell models remain important goals for future work.

Several limitations of the present study warrant consideration. The use of cancer-derived cell lines (HL60 and HepG2) rather than primary human cells introduces inherent constraints on extrapolation to normal human physiology, as these lines possess dysregulated cell cycle control and lack three-dimensional tissue architecture (Arzumanian et al., 2021; Birnie, 1988). Translation to occupational and environmental exposure scenarios involves additional uncertainty: standard 100-fold safety factors are applied when interpreting results (Petersen et al., 2025), and while the lower end of our concentration range (0.1–10 μM) reflects environmentally relevant exposures, the approach cannot fully account for absorption, distribution, metabolism, and excretion dynamics in intact biological systems (Kim et al., 2024). The 1-h exposure duration, while appropriate for acute genotoxicity screening and consistent with prior publications from our group, may not capture effects arising from chronic low-dose exposures characteristic of real-world scenarios. The absence of formal ROS fluorescence quantification and cell morphology imaging—approaches such as DCFH-DA staining that have provided valuable mechanistic context in comparable pesticide studies—represents a further limitation that future investigations should address. Such studies should also incorporate *in vivo* validation, transcriptomic and proteomic analyses to identify disrupted pathways, and ideally primary human cells or organoid models to improve physiological relevance and extend the present findings. Additionally, the inclusion of a pure glyphosate arm would strengthen the comparative analysis. However, our prior publications (Nagy et al., 2021; Nagy et al., 2019) in the same experimental system directly compare pure glyphosate with the same three commercial formulations tested here, consistently demonstrating that glyphosate alone produces minimal cytotoxicity and genotoxicity at the concentrations where formulations show significant effects. To avoid redundancy with already published data from our group and to remain within the scope of this study (formulation versus co-formulant comparison), pure glyphosate was not included.

Despite these constraints, HL60 and HepG2 remain well-validated models for initial comparative toxicological screening, offering reproducibility, extensive literature characterisation, and representation of two major human target tissues (Allen et al., 2005). The use of 2 cell types from distinct tissue origins enabled identification of cell-type-specific genotoxic responses and provides a broader basis for human health impact assessment than single-cell-line approaches. These findings therefore serve as a foundation for more mechanistically detailed and physiologically relevant studies, in alignment with the 3 R s principle of toxicology testing (ITR-Laboratories, 2026).

## Conclusion

This study demonstrates that commercial GBH formulations exhibit cell type- and formulation-specific cytotoxic and genotoxic profiles that differ substantially from those of the active ingredient alone. Roundup Mega was the most genotoxic agent in HL60 cells, and Glyfos showed the earliest consistent genotoxic effects in HepG2 cells. The co-formulants demonstrated limited genotoxicity relative to the full formulations, suggesting that synergistic interactions between glyphosate and adjuvants—rather than adjuvants alone—underlie the enhanced genotoxicity of commercial products. The consistent occurrence of DNA damage at sub-cytotoxic concentrations in our studies provides further evidence for the genotoxic potential of GBHs, as well as highlights that more robust formulation-specific genotoxicity testing should be integrated into pesticide risk assessment to better protect human health.

## Data Availability

The original contributions presented in the study, and the summarised statistical outputs supporting the findings of this study — including mean differences, p-values, and significance classifications for all cytotoxicity and genotoxicity endpoints across both cell lines, are included in the article/Supplementary Material. The underlying raw data (raw comet images and numerical scoring files) are not publicly available due to confidentiality. Further inquiries can be directed to the corresponding author.
