# Supplementary material for: Comparative cytotoxicity and genotoxicity of commercial glyphosate-based herbicide formulations and co-formulants in human leukocyte and hepatocyte cell lines
Source: Front Toxicol. 2026 Jul 3;8:1770738. doi: 10.3389/ftox.2026.1770738 (PMC13375184; doi:10.3389/ftox.2026.1770738)
Supplement: Supplementary file 4 [file Table4.docx]

***Supplemental table S4.*** *Mean differences and significance values for genotoxic effects of glyphosate-based formulations and co-formulants in HepG2 cells in* *comparison to untreated control*


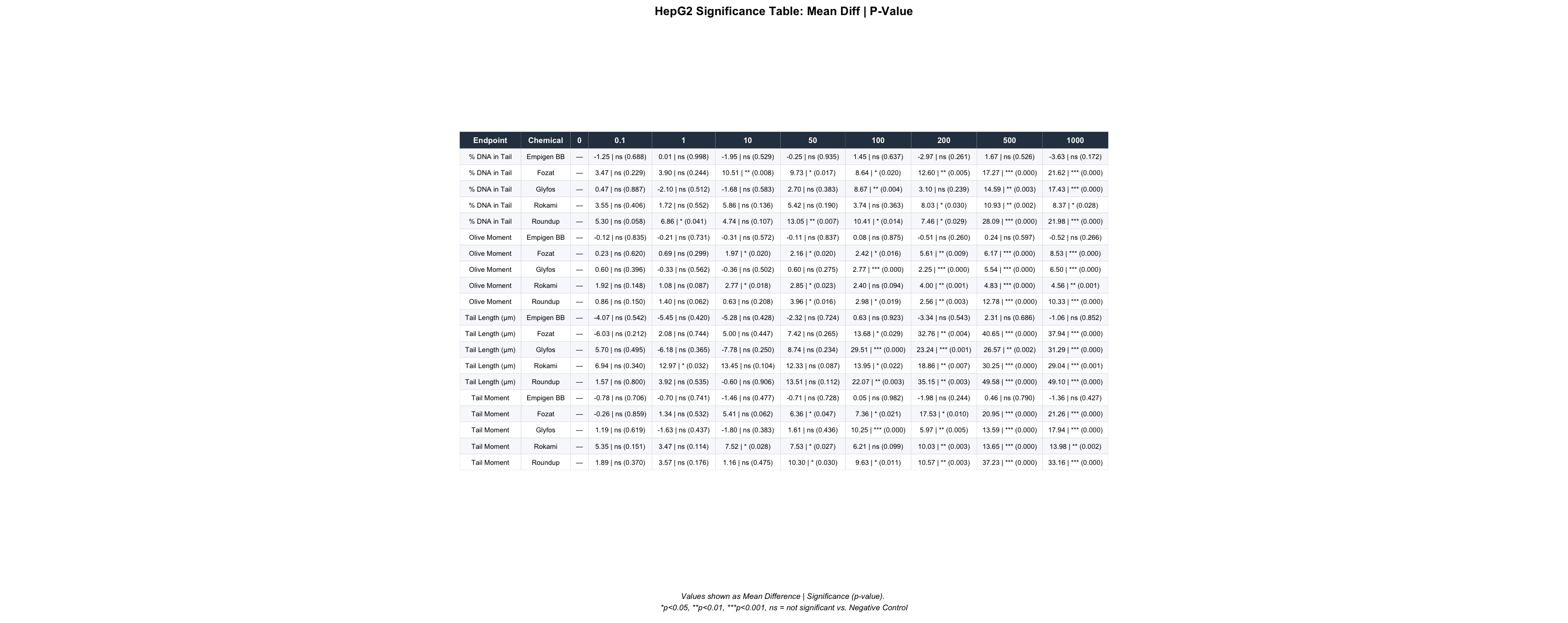


*Values shown as Mean Difference | Significance (p−value).* **p<0.05, **p<0.01, ***p<0.001, ns = not significant vs. Negative Control*
