# Supplementary material for: Comparative cytotoxicity and genotoxicity of commercial glyphosate-based herbicide formulations and co-formulants in human leukocyte and hepatocyte cell lines
Source: Front Toxicol. 2026 Jul 3;8:1770738. doi: 10.3389/ftox.2026.1770738 (PMC13375184; doi:10.3389/ftox.2026.1770738)
Supplement: Supplementary file 5 [file Image1.pdf]

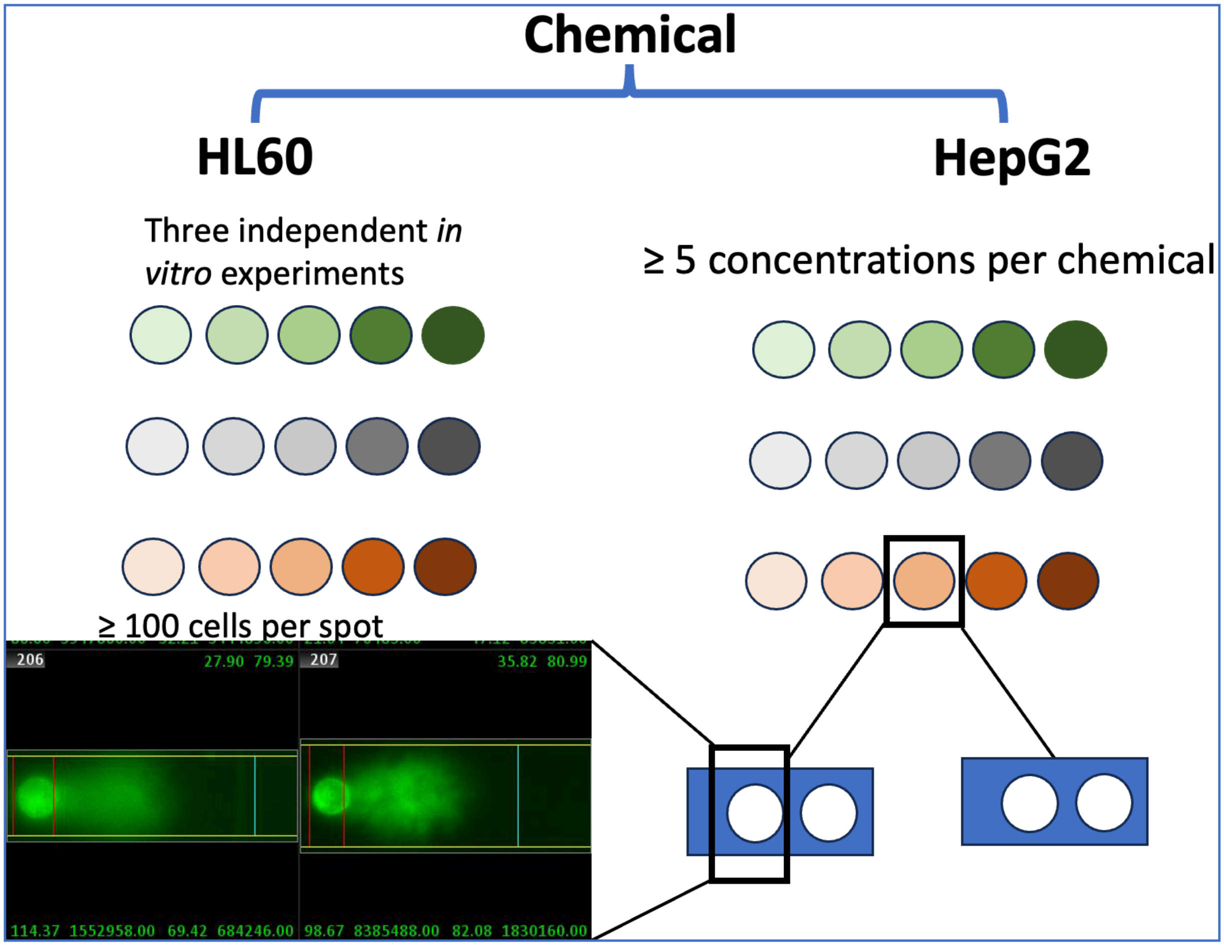

Supplemental figure S1. Integrated experimental framework for cytotoxicity and genotoxicity profiling. *HL60 and HepG2 cell lines were exposed to a range of chemical concentrations. Dosages for the subsequent comet assay were determined based on preliminary cytotoxicity findings. To ensure technical precision, duplicate samples were processed per experiment, with slides strategically placed at the front and rear of the electrophoresis chamber. Data integrity was maintained by including only those samples with a minimum count of 100 cells per treatment group per experimental run.*
